# The role of inflammation in the effects of peer victimisation and stressful life events on mental health in childhood

**DOI:** 10.1016/j.bbih.2023.100695

**Published:** 2023-10-14

**Authors:** Ellie Roberts, Marta Francesconi, Eirini Flouri

**Affiliations:** a Department of Arts and Sciences, University College London, Malet Place, London, NW1 6AP, UK; b Department of Psychology and Human Development, University College London, 25 Woburn Square, London, WC1H 0AA, UK

**Keywords:** Peer victimisation, Inflammation, Stress, Peer problems, ALSPAC

## Abstract

**Background:**

Peer victimisation represents a salient stressor during childhood. However, studies investigating the mechanism of its impact on children's mental health typically examine socio-cognitive factors as mediators. The current study sought to provide novel insight through testing a potential biological mechanism, inflammation. It also tested for pathway-specific effects by comparing how inflammation may mediate the effect of peer victimisation and that of another important stressor in childhood: adverse life events.

**Method:**

Data from 4,583 participants of the Avon Longitudinal Study of Parents and Children (ALSPAC) were used. Path analysis was carried out to investigate whether inflammation (IL-6 and CRP) at age 9 years mediates the effect of peer victimisation and stressful life events at age 8 years on internalising (peer and emotional) or externalising (hyperactivity and conduct) problems (measured at age 11 years), both before and after adjustment for potential confounders.

**Results:**

IL-6 partially mediated the effect of peer victimisation on peer problems, even after adjustment for potential confounders. Inflammation did not mediate the effect of stressful life events on either type of internalising problems. Neither stressor predicted externalising problems via inflammation.

**Conclusion:**

We did not find evidence that inflammation mediates the effect of stressful life events on mental health in childhood when they are considered alongside experiences of peer victimisation. Inflammation may already represent a form of biological embedding of peer victimisation in the early years.

## Highlights

• Both peer victimisation and stressful life events predict child ill-mental health.
• IL-6 partially mediates the effect of peer victimisation on peer problems.
• The effect of life events on mental health is not mediated by inflammation.
• Neither stressor predicts externalising problems via inflammation.

## Lay summary

This study explored how being bullied by peers and experiencing stressful life events can affect children's mental health. We looked at whether inflammation, a biological process in the body, might explain the effects of these experiences on child mental health. We used data from almost 5,000 children and tested whether inflammation at age 9 could explain why these two experiences earlier in childhood (ages 7–8 years) may be related to mental health problems at the end of primary school (age 11).

The results showed that inflammation might partly explain the link between being bullied and one aspect of mental health at age 11, having problems with peers. However, inflammation did not seem to play a role in the connection between stressful life events, when considered alongside peer victimisation, and any type of mental health problems at age 11. Thus, inflammation may be an important factor in explaining the effect of peer victimisation, but not other types of stressors, on some aspects of child mental health. Inflammation therefore may be a mechanism explaining why children bullied by their peers experience social difficulties. Other types of stressors did not seem to have the same biological effect.

## 1 Introduction

Peer victimisation refers to a specific type of bullying that is among peers and represents a major stressor during childhood (Arseneault, 2018; Perren et al., 2013). It can be overt or relational, although the two frequently co-occur, with overt victimisation being more common before adolescence and usually having more serious impacts (Casper and Card, 2017). But irrespective of its type, peer victimisation appears to be a very salient risk factor for poor mental health because even when it is transient it can produce long-term effects (Klomek et al., 2011; Ttofi et al., 2011). For example, Lereya et al. (2015) found that the effect of childhood peer victimisation on mental health in adulthood was greater than that of maltreatment by a caregiver. An earlier study had shown that half of children who had experienced peer victimisation reported that they were still victimised 3 years later (Scholte et al., 2007), suggesting that peer victimisation in childhood is a major stressor and often a chronic one as well. Importantly, monozygotic twin studies have shown that it is largely an environmental risk factor. For example, an early study by Arseneault et al. (2008) demonstrated that monozygotic twins who were bullied at age 7 suffered more emotional problems at age 10 compared to their co-twins that had not, even after accounting for emotional problems prior to bullying.

Given its salience, peer victimisation has also attracted much research in how its mental health effects on children are produced. Most of this research has focused on socio-cognitive mechanisms. For example, hostile attributions, poor interpersonal skills, low social-perspective awareness, and poor coping strategies have all been found to mediate the effect of peer victimisation on internalising and externalising behaviours in children and adolescents (Hoglund and Leadbeater, 2007; Perren et al., 2013; Singh and Bussey, 2011). Biological processes, particularly increases in inflammatory activity, however have largely been ignored. Yet, inflammation may be a plausible mechanism given the evidence for the effect of early stressors on inflammatory response in both children and adults (Baumeister et al., 2016; Slopen et al., 2013a). Case-control studies, for instance, have revealed that children who had experienced maltreatment had higher levels of inflammation in adulthood compared to children who had not (Danese et al., 2007; Entringer et al., 2020), with some studies showing such long-term effects specifically for peer victimisation in childhood (Copeland et al., 2014; Takizawa et al., 2015). Inflammation has in turn been associated with poor mental health. Linkas et al. (2022), for instance, observed higher levels of inflammation in adolescents with psychological distress compared to those without. Although the direction of causality is unclear given the nature of the data available, it appears that poor mental health may be the outcome rather than the cause of inflammation. For example, a prospective study by Khandaker et al. (2014) found that inflammation at age 9 years could predict depressive symptoms at age 18 years. Such patterns have also been observed during childhood (Mitchell and Goldstein, 2014) whereby, in a more recent study using the same dataset, inflammation at age 9 years predicted internalising problems at age 11 years (Flouri et al., 2019).

### 1.1 The current study

Despite much interest in the role of inflammation in explaining the impact of stressors on mental health, no study has investigated whether inflammation mediates the effect of a salient stressor in childhood, peer victimisation, on children's mental health (internalising and externalising problems). Furthermore, there is a need to understand the specificity of such a biological mechanism in childhood, for both stressor and mental health outcome. The current study was designed to fill both gaps. It sought to uncover whether inflammation [interleukin 6 (IL-6) and C-reactive protein (CRP)] mediates the effect of peer victimisation on mental health in childhood in the general population. It also investigated whether peer victimisation and stressful life events, contemporaneously measured in middle childhood, differ in the size of both their direct and indirect (via inflammation) effects on mental health later in childhood. In this study, therefore, measures of both peer victimisation and stressful life events in middle childhood were considered alongside one another, enabling each stressor to compare with and control for the other. To ensure that the impact of both peer victimisation and stressful life events in middle childhood was not confounded by earlier exposure to stressors and family adversity, experience of stressful life events from birth until middle childhood (see Measures) was also controlled for.

## 2 Method

### 2.1 Participants

The Avon Longitudinal Study of Parents and Children (ALSPAC) is an ongoing prospective cohort study, assessing biological, psychological, social, and other developmental exposures during pregnancy and after birth which may affect the development and wellbeing of the child (Boyd et al., 2013; http://www.bristol.ac.uk/alspac/researchers/our-data for details of all the data that is available). The ALSPAC recruited 14,541 women in Bristol, UK from April 1991 to December 1992. From the first pregnancy trimester, parents completed questionnaires about both their own and their child's wellbeing. From age 7 onwards, children were annually invited to attend assessments at clinics, during which they took part in face-to-face interviews and underwent physical and psychological tests (Fraser et al., 2013). At the age of 12 months, 13,988 children were still alive, and the development of these children has since been followed (Golding & ALSPAC Study Team, 2004). Of the original 14,541 initial pregnancies, 338 were from a woman who had already enrolled with a previous pregnancy, meaning 14,203 unique mothers were initially enrolled in the study. As a result of the additional phases of recruitment, a further 630 women who did not enrol originally have provided data since their child was 7 years of age, increasing the total number to 15,447 pregnancies and a subsequent 14,901 children alive at 12 months of age (Fraser et al., 2013). Consent for biological samples has been collected in accordance with the Human Tissue Act (2004). Ethical approval for the study was obtained from the ALSPAC Ethics and Law Committee and Local Research Ethics Committees (further details can be found at https://www.bristol.ac.uk/alspac/). The current study's analytic sample size (N = 4,583) comprises children who had valid data on inflammatory markers at age 9 (the only time in childhood they were measured in ALSPAC) and did not report an infection at the time of blood collection or during the preceding week.

### 2.2 Measures

#### 2.2.1 Stressful life events (ages 7–8)

Stressful life events were measured at age about 8 years using a mother-completed checklist comprising a list of 17 potentially upsetting events for the child that may have occurred since the child's 7th birthday. If the event had occurred, the response was coded such that the child was either “very upset”, “quite upset”, “a bit upset” or “was not upset”. Each event was scored dichotomously as having occurred or not (score of 1 or 0, respectively), regardless of the degree of upset. Scores were then summed to produce a total number of stressful life events experienced by the child from age 7 to age 8.

#### 2.2.2 Peer victimisation (age 8)

Peer victimisation was assessed at age 8 using the child-reported Bullying and Friendship Interview Schedule (Hamburger et al., 2011), which has demonstrated high predictive ability and inter-rater reliability (Zwierzynska et al., 2013). Children were asked to rate their experiences of peer victimisation over the past six months using five questions for overt victimisation (e.g., being hit or beaten up) and four for relational victimisation (e.g., being socially excluded). If children answered ‘yes’ to a victimisation event having occurred, they were asked how frequently it had happened over the past six months, as follows: 1 to 3 times (infrequently), ≥4 times (frequently), or at least once a week (very frequently). Overt or relational victimisation was deemed present in our study if children had experienced it frequently or very frequently (Wolke et al., 2000). In view of the salience of overt victimisation for poor outcomes in our developmental stage, the exposure for our main analysis is whether an experience of overt pure victimisation (hereafter “peer victimisation”) occurred or not.

#### 2.2.3 Inflammation (age 9)

Inflammation was measured by interleukin 6 (IL-6) and C-reactive protein (CRP) levels at age 9, obtained from blood samples during clinic visits (see also Ji et al., 2022). Blood samples were collected from non-fasting participants before being immediately spun and frozen at −80 °C, with no evidence of freeze-thaw cycles during storage. IL-6 (pg/mL) was measured by enzyme-linked immunosorbent assay (R&D Systems, Abingdon, UK) and high-sensitivity CRP (mg/L) was measured by automated particle-enhanced immunoturbidimetric assay (Roche, UK). All interassay coefficients of variation were less than 5%. IL-6 and CRP measures were log-transformed in this analysis to correct for skewness.

#### 2.2.4 Internalising and externalising problems (age 11)

Internalising and externalising problems were measured at age 11 using the mother-completed Strengths and Difficulties Questionnaire (SDQ) (Goodman et al., 2010), which has good reliability and validity in various population samples (Goodman, 2001). The SDQ contains 20 items relating to child behaviours over the past 6 months, forming four subscales: hyperactivity, emotional symptoms, conduct problems, and peer problems. The items are scored on a 3-point scale (0 = ‘not true’, 1 = ‘somewhat true’, 2 = ‘certainly true’), with higher scores implying the presence of more severe difficulties. Internalising problems are the combination of the items of the emotional and peer problems subscales, while externalising problems comprise the combined scores of the hyperactivity and conduct problems subscales. The internalising and externalising problems scales therefore each comprise 10 items, with scores ranging from 0 to 20.

#### 2.2.5 Confounders

Several confounders associated with stress exposure, peer victimisation, inflammation and mental health were controlled for. These included sex (at birth) (Carbone-Lopez et al., 2010; Gutman and McMaster, 2020; Martinez-Muniz and Wood, 2020), being overweight (at age 9) (Bacchini et al., 2015; Khanna et al., 2022; Luppino et al., 2010), ethnicity (at birth) (Bains and Gutman, 2021; Schmeer and Tarrence, 2018; Xu et al., 2020a), socioeconomic status (measured by maternal education and paternal social class) (Businelle et al., 2014; Jansen et al., 2012; Muscatell et al., 2020; Reiss et al., 2019), and maternal depression (Conron et al., 2009; Henry et al., 2020; Nomaguchi and Fettro, 2020; Ulmer-Yaniv et al., 2018). Sex and ethnicity were dichotomously coded: male and female, white and non-white, respectively. Being overweight was determined with the International Obesity Task Force (IOTF) sex- and age-specific cut-offs for body mass index (BMI) (Cole et al., 2000). Maternal education (attainment) was measured at 32 weeks of pregnancy as one of the following qualifications: CSE, vocational, O level, A level, and degree, with higher values representing higher levels of education. Paternal social class was also measured at 32 weeks of pregnancy and divided into categories of: I, II, III (non-manual), III (manual), IV, and V, with higher values therefore representing lower levels of social class. Maternal depression was measured using the Edinburgh Postnatal Depression Scale (EPDS) at 32 weeks of pregnancy, with a higher score indicating a greater level of depression (Cox et al., 1987). As explained, we also adjusted for history of exposure to stress prior to the measurement of our main risk factors at age 7–8 years. In ALSPAC this was measured using a checklist of 43 life events, completed by the mother when the child was 21 months (covering events since the child was 8 months), 33 months (covering events since the child was 18 months), 47 months (covering events since the child was 33 months), 61 months (covering events since the child was 47 months), 73 months (covering events since the child was 61 months) (Barnett et al., 1983). For our analysis, we derived a life events score at ages 1–6 years by calculating whether each of the 43 events ever occurred between 8 and 73 months.

### 2.3 Analytic strategy

Analyses were performed in SPSS and AMOS version 29. Descriptive statistics were used to summarise the sample. Bivariate correlations, using Pearson's correlation coefficients, were calculated between the main variables (i.e., exposures, mediators, and outcomes) of peer victimisation, stressful life events, inflammatory markers, and internalising and externalising problems. Regression imputation with maximum likelihood estimates was used to handle missing data. Path analysis models were fitted in AMOS to test mediation paths from both peer victimisation and life events (which were mutually adjusted in all models, as explained) via both CRP and IL-6, before and after controlling for confounders. Models were fitted separately for each of the four SDQ subscales (emotional symptoms, peer problems, hyperactivity and conduct problems) as well as for internalising problems (emotional symptoms and peer problems) and externalising problems (hyperactivity and conduct problems).

## 3 Results

### 3.1 Descriptive statistics

The descriptive statistics of the sample are shown in Table 1. The sample was mostly of white ethnicity (96%) but almost equally divided by sex (49% female). The most common level of maternal education was O level (35.1%) and the most common paternal social class was II (36.4%). Most children were not involved in overt (65.8%) or relational (83.2%) bullying either as a bully or as a victim. However, those who were involved reported most frequently that they were a pure victim, which was observed for both overt (27.4%) and relational bullying (14.3%). The most endorsed SDQ subscale was hyperactivity (M=2.71), as expected.

**Table 1:** Descriptive statistics of all variables in the sample (N = 4,583).

Categorical variables		
	**N**	**%**
Sex (Female)	2243	49.0
Ethnicity (White)	3983	96.0
**Maternal education**		
CSE	552	13.1
Vocational	330	7.8
O level	1476	35.1
A level	1135	27.0
Degree	711	16.9
**Paternal social class**		
I	490	12.6
II	1412	36.4
III (non-manual)	478	12.3
III (manual)	1102	28.4
IV	309	8.0
V	84	2.2
**Overt bullying status**		
Pure bully	42	1.1
Pure victim	1047	27.4
Bully-victim	220	5.7
Neutral	2518	65.8
**Relational bullying status**		
Pure bully	26	0.7
Pure victim	538	14.3
Bully-victim	66	1.8
Neutral	3123	83.2
**Continuous variables**		
	**N**	**Mean (SD)**
Maternal stressful life events	2534	11.59 (4.35)
Stressful life events (child-specific), age 7-8	2856	2.03 (1.21)
Interleukin 6 (pg/mL)	4583	1.21 (1.47)
C-Reactive protein (mg/L)	4583	0.62 (1.93)
BMI	4531	17.57 (2.76)
Hyperactivity	3630	2.71 (2.22)
Emotional symptoms	3631	1.34 (1.62)
Conduct problems	3639	1.16 (1.42)
Peer problems	3640	1.05 (1.50)
Maternal depression	4137	6.70 (4.86)

### 3.2 Bivariate correlations

Bivariate correlations using Pearson's correlation coefficients were calculated between the main variables. The results can be seen in Table 2. Peer victimisation correlated with stressful life events (p < .01). It was also most strongly associated with peer problems, followed by hyperactivity, conduct problems and emotional problems. Stressful life events most strongly correlated with emotional problems, followed by peer problems, hyperactivity and conduct problems. Peer victimisation correlated with both IL-6 (p < .05) and CRP (p < .05), but more strongly with IL-6, while stressful life events only correlated with IL-6 (p <.05). IL-6 correlated with peer problems (p < .01) and emotional problems (p < .05) but did not significantly correlate with any externalising problems. CRP correlated with emotional problems (p < .01) and was negatively associated with hyperactivity (p < .05), but did not significantly correlate with either peer or conduct problems.

**Table 2:** Bivariate correlations between the main variables.

	1.	2.	3.	4.	5.	6.	7.	8.
**1. Victim**	1							
**2. Life events**	.072**	1						
**3. IL-6**	.040*	.039*	1					
**4. CRP**	.034*	.007	.452**	1				
**5. Hyper**	.106**	.065**	−.018	−.040*	1			
**6. Emotion**	.053**	.105**	.041*	.053**	.285**	1		
**7. Conduct**	.064**	.056**	.030	.008	.490**	.291**	1	
**8. Peer**	.110**	.068**	.052**	.028	.275**	.362**	.285**	1

Victim = Peer victimisation; Hyper = Hyperactivity; Emotion = Emotional problems; Conduct = Conduct problems; Peer = Peer problems (*p < .05; **p < .01).

### 3.3 Path analysis

Table 3 summarises the regression coefficients, before and after adjusting for confounders, for models that were significant after adjustment (see Supplementary Material for the results of all other models fitted). As can be seen, there was a significant indirect effect of peer victimisation on peer problems via IL-6 (unadjusted model: b = 0.006, 95% CI [0.002, 0.015], p < .01), which was robust to adjustment for confounders (adjusted model: b = 0.003, 95% CI [0.000, 0.010], p < .05). The effect of peer victimisation on peer problems therefore was partially mediated by IL-6. All other mediation models were not significant, either before or after adjustment.

**Table 3:** Unadjusted and adjusted unstandardised regression coefficients of the model testing mediation of the effect of peer victimisation on peer problems by IL-6.

Peer Victimisation (PV) → IL-6 → Peer Problems (PP)			
	b	SE	95% CI
**Unadjusted model**			
PV → PP	.383***	.048	.275, .488
PV → IL-6	.035**	.013	.008, .061
IL-6 → PP	.182***	.053	.076, .289
Indirect effect	.006**	.003	.002, .015
Total effect	.389***	.054	.283, .494
***Adjusted model***			
PV → PP	.339***	.047	.231, .443
PV → IL-6	.028*	.013	.003, .053
IL-6 → PP	.123*	.054	.020, .229
Indirect effect	.003*	.002	.000, .010
Total effect	.343***	.054	.236, .447

(*p < .05; **p < .01; ***p < .001).

The total effect of peer victimisation on peer problems (adjusted model: standardised b = .105, 95% CI [0.073, 0.137], p < .001) and the total effect of stressful life events on peer problems (adjusted model: standardised b = .050, 95% CI [0.019, 0.083], p < .001) were compared using a z-test for differences between regression coefficients (Clogg et al., 1995; Paternoster et al., 1998). The total effect of peer victimisation was significantly larger (p < .05). The direct effect of peer victimisation (adjusted model: standardised b = .104, 95% CI [0.072, 0.135], p < .001) was also significantly larger (p < .05) than that of stressful life events (adjusted model: standardised b = .050, 95% CI [0.018, 0.082], p < .001).

We then determined the size of the mediating effect we found by calculating the proportion of the total effect that can be explained by the indirect effect: the proportion mediated (PM) (Mackinnon et al., 2007), which performs well in sample sizes over 500 (Fairchild and McQuillin, 2010). The PM represents a value between 0 and 1, whereby a value of 1 indicates that the entire effect of the predictor variable on the outcome variable can be attributed to the mediating variable, whereas a value of 0 implies that none of the effect can. The PM by IL-6 in the effect of peer victimisation on peer problems in our study was 0.009.

## 4 Discussion

Using longitudinal data from a large general-population sample in the UK, this study provided clear evidence for the unique role of overt peer victimisation in mental health, particularly peer relationship problems, in childhood. It also shed light into a biological process that may explain some of its impact. It showed that overt peer victimisation had a larger effect than exposure to stressful life events on peer problems, and that this effect was partially mediated by IL-6. Importantly, the indirect effect of overt peer victimisation via IL-6 was robust to adjustment for other stressors experienced during childhood in addition to several potential confounding factors, but it was also very specific: inflammation could only explain part of the effect of overt peer victimisation only on peer problems. Stressful life events were, as expected, related to mental health problems in children, but when considered alongside overt peer victimisation they did not have an indirect effect on mental health via inflammation. Together, these findings indicate that, when exposure to stressors in childhood is comprehensively considered, inflammation has a very specific role in explaining the effect of specific stressors on specific mental health outcomes in children in the general population. If the associations found in this study are causal, they suggest that peer victimisation leads to social difficulties in childhood partly by producing an inflammatory response.

It is important to note that inflammation did not mediate the pathway from either stressor to externalising problems in children, which echoes earlier evidence that it is not generally related to such behaviours in children (Flouri et al., 2019). Nevertheless, our finding that inflammation did not mediate the effect of stressful life events on internalising problems contradicts previous research in the same sample (Flouri et al., 2019). In the current study, however, stressful life events were considered alongside overt peer victimisation, the stressor producing the effect modelled here. Another reason for the discrepancy may be differences in the operationalisation of stressful life events, as Flouri et al. (2019) did not measure stressful life events as experienced by the child directly, but those that were experienced by the mother and the family. Therefore, the current study incorporates a more accurate measure of stressor exposure by considering general stressful events experienced directly by the child, a specific and salient stressor (overt peer victimisation), and stressful life events experienced by the family since the child's birth.

Our study also adds to the evidence about the salience of peer victimisation in childhood. Research has long established that being bullied in childhood is associated with both higher levels of inflammation and social problems in adulthood (Lidberg et al., 2023; Takizawa et al., 2014, 2015). The current study showed that peer victimisation, even when considered alongside other stressful life events at the level of both the child and the family, can produce such biological effects from childhood. Studies in mice have revealed that social defeat stress early in life increases IL-6 levels (Xu et al., 2020b), which subsequently result in social avoidance behaviours (Niraula et al., 2019). The current findings indicate that similar mechanisms may explain the impact of peer victimisation on social development in human childhood, whereby inflammation embeds the effects of peer victimisation at a biological level, resulting in long-term difficulties interacting and integrating with others. Such a mechanism would, in turn, complement recent findings that peer victimisation is associated with structural and functional brain changes in childhood and adolescence, including those linked to hypersensitivity to social exclusion and social monitoring (Cubillo, 2022). Future research could investigate whether peer victimisation produces such changes via inflammation.

However, these findings should be seen in the light of an important limitation of the study, namely its observational design, which precludes causal inferences. For example, peer problems could be the determinant and not the outcome of overt peer victimisation or inflammation. Indeed, peer and other internalising problems have been found to predict heightened levels of inflammation (Flouri et al., 2020; Slopen et al., 2013b). However, Gimeno et al. (2009) compared the effect of internalising problems on inflammation, and that of inflammation on internalising problems. While the relationship was found to be reciprocal, the effect of inflammation on internalising problems was greater. Furthermore, Flouri et al. (2019) investigated the potential reciprocal relationship between stressful events and internalising problems and found that stressful events could predict internalising problems and not the other way around.

## 5 Conclusion

The current study demonstrated that the effect of overt peer victimisation on peer problems in childhood was partially mediated by IL-6. Peer victimisation can therefore affect children's social development at a biological level. We do acknowledge however that the size of the indirect effect via inflammation was very small, suggesting that other mechanisms are at play. Furthermore, when considered alongside overt peer victimisation, stressful life events did not have an indirect effect on mental health via inflammation. These findings highlight the need to compare the effects, and associated underlying mechanisms, of different stressors on child mental health, as these may be very specific.

## Declaration of competing interest

None

## Data Availability

The authors do not have permission to share data.
