# Supplementary material for: The role of inflammation in the effects of peer victimisation and stressful life events on mental health in childhood
Source: Brain Behav Immun Health. 2023 Oct 14;34:100695. doi: 10.1016/j.bbih.2023.100695 (PMC10641088; doi:10.1016/j.bbih.2023.100695)
Supplement: Multimedia component 1 [file mmc1.docx]

**Supplementary material**

Table S1. Unadjusted and adjusted unstandardised regression coefficients (life events 🡪 IL-6 🡪 peer problems)

| **Life Events (LE) 🡪 IL-6 🡪 Peer Problems (PP)** | | | |
| --- | --- | --- | --- |
|  | **B** | **SE** | **95% CI** |
| ***Unadjusted model*** | | | |
| LE 🡪 PP | .097*** | .020 | .050, .143 |
| LE 🡪 IL-6 | .017** | .006 | .006, .029 |
| IL-6 🡪 PP | .182*** | .053 | .076, .289 |
| Indirect effect | .003*** | .001 | .001, .007 |
| Total effect | .100*** | .024 | .053, .145 |
| ***Adjusted model*** | | | |
| LE 🡪 PP | .069*** | .020 | .025, .115 |
| LE 🡪 IL-6 | .006 | .005 | -.005, .017 |
| IL-6 🡪 PP | .123* | .054 | .020, .229 |
| Indirect effect | .001 | .001 | .000, .003 |
| Total effect | .070*** | .023 | .025, .115 |

Table S2. Unadjusted and adjusted unstandardised regression coefficients (peer victimisation 🡪 CRP 🡪 peer problems)

| **Peer Victimisation (PV) 🡪 CRP 🡪 Peer Problems (PP)** | | | |
| --- | --- | --- | --- |
|  | **B** | **SE** | **95% CI** |
| ***Unadjusted model*** | | | |
| PV 🡪 PP | .355*** | .048 | .246, .457 |
| PV 🡪 CRP | .039* | .018 | .000, .076 |
| CRP 🡪 PP | .079* | .039 | -.003, .164 |
| Indirect effect | .003* | .002 | .000, .011 |
| Total effect | .358*** | .054 | .249, .461 |
| ***Adjusted model*** | | | |
| PV 🡪 PP | .341*** | .047 | .234, .446 |
| PV 🡪 CRP | .028 | .016 | -.004, .061 |
| CRP 🡪 PP | .015 | .044 | -.075, .108 |
| Indirect effect | .000 | .002 | -.002, .005 |
| Total effect | .342*** | .054 | .235, .446 |

Table S3. Unadjusted and adjusted unstandardised regression coefficients (life events 🡪 CRP 🡪 peer problems)

| **Life Events (LE) 🡪 CRP 🡪 Peer Problems (PP)** | | | |
| --- | --- | --- | --- |
|  | **b** | **SE** | **95% CI** |
| ***Unadjusted model*** | | | |
| LE 🡪 PP | .086*** | .020 | .039, .131 |
| LE 🡪 CRP | .004 | .008 | -.012, .019 |
| CRP 🡪 PP | .079* | .039 | -.003, .164 |
| Indirect effect | .000 | .001 | -.001, .002 |
| Total effect | .086*** | .024 | .040, .132 |
| ***Adjusted model*** | | | |
| LE 🡪 PP | .070*** | .020 | .026, .116 |
| LE 🡪 CRP | -.016* | .007 | -.030, -.003 |
| CRP 🡪 PP | .015 | .044 | -.075, .108 |
| Indirect effect | .000 | .001 | -.002, .001 |
| Total effect | .071*** | .023 | .026, .116 |

Table S4. Unadjusted and adjusted unstandardised regression coefficients (peer victimisation 🡪 IL-6 🡪 emotional problems)

| **Peer Victimisation (PV) 🡪 IL-6 🡪 Emotional Problems (EP)** | | | |
| --- | --- | --- | --- |
|  | **b** | **SE** | **95% CI** |
| ***Unadjusted model*** | | | |
| PV 🡪 EP | .168*** | .052 | .062, .273 |
| PV 🡪 IL-6 | .035** | .013 | .008, .061 |
| IL-6 🡪 EP | .154** | .058 | .041, .268 |
| Indirect effect | .005** | .003 | .001, .013 |
| Total effect | .174** | .055 | .065, .277 |
| ***Adjusted model*** | | | |
| PV 🡪 EP | .134** | .051 | .030, .237 |
| PV 🡪 IL-6 | .028* | .013 | .003, .053 |
| IL-6 🡪 EP | .041 | .058 | -.073, .152 |
| Indirect effect | .001 | .002 | -.002, .006 |
| Total effect | .135** | .053 | .031, .238 |

Table S5. Unadjusted and adjusted unstandardised regression coefficients (life events 🡪 IL-6 🡪 emotional problems)

| **Life Events (LE) 🡪 IL-6 🡪 Emotional Problems (EP)** | | | |
| --- | --- | --- | --- |
|  | **b** | **SE** | **95% CI** |
| ***Unadjusted model*** | | | |
| LE 🡪 EP | .184*** | .022 | .133, .241 |
| LE 🡪 IL-6 | .017** | .006 | .006, .029 |
| IL-6 🡪 EP | .154** | .058 | .041, .268 |
| Indirect effect | .003** | .001 | .001, .006 |
| Total effect | .187*** | .027 | .135, .242 |
| ***Adjusted model*** | | | |
| LE 🡪 EP | .136*** | .021 | .085, .189 |
| LE 🡪 IL-6 | .006 | .005 | .003, .053 |
| IL-6 🡪 EP | .041 | .058 | -.073, .152 |
| Indirect effect | .000 | .001 | .000, .002 |
| Total effect | .136*** | .026 | .086, .190 |

Table S6. Unadjusted and adjusted unstandardised regression coefficients (peer victimisation 🡪 CRP 🡪 emotional problems)

| **Peer Victimisation (PV) 🡪 CRP 🡪 Emotional Problems (EP)** | | | |
| --- | --- | --- | --- |
|  | **b** | **SE** | **95% CI** |
| ***Unadjusted model*** | | | |
| PV 🡪 EP | .171*** | .052 | .065, .276 |
| PV 🡪 CRP | .044* | .018 | .006, .082 |
| CRP 🡪 EP | .169*** | .043 | .084, .258 |
| Indirect effect | .007* | .004 | .001, .017 |
| Total effect | .179*** | .054 | .070, .282 |
| ***Adjusted model*** | | | |
| PV 🡪 EP | .135** | .051 | .032, .239 |
| PV 🡪 CRP | .030 | .016 | -.003, .063 |
| CRP 🡪 EP | .094* | .047 | .001, .187 |
| Indirect effect | .003 | .002 | .000, .009 |
| Total effect | .138** | .053 | .034, .241 |

Table S7. Unadjusted and adjusted unstandardised regression coefficients (life events 🡪 CRP 🡪 emotional problems)

| **Life Events (LE) 🡪 CRP 🡪 Emotional Problems (EP)** | | | |
| --- | --- | --- | --- |
|  | **b** | **SE** | **95% CI** |
| ***Unadjusted model*** | | | |
| LE 🡪 EP | .187*** | .022 | .135, .241 |
| LE 🡪 CRP | .003 | .008 | -.013, .019 |
| CRP 🡪 EP | .169*** | .043 | .084, .258 |
| Indirect effect | .001 | .001 | -.002, .004 |
| Total effect | .187*** | .027 | .135, .242 |
| ***Adjusted model*** | | | |
| LE 🡪 EP | .137*** | .021 | .087, .192 |
| LE 🡪 CRP | -.017* | .007 | -.031, -.003 |
| CRP 🡪 EP | .094* | .047 | .001, .187 |
| Indirect effect | -.002* | .001 | -.005, .000 |
| Total effect | .139*** | .026 | .086, .190 |

Table S8. Unadjusted and adjusted unstandardised regression coefficients (peer victimisation 🡪 IL-6 🡪 internalising)

| **Peer Victimisation (PV) 🡪 IL-6 🡪 Internalising** | | | |
| --- | --- | --- | --- |
|  | **b** | **SE** | **95% CI** |
| ***Unadjusted model*** | | | |
| PV 🡪 Internalising | .558*** | .082 | .381, .727 |
| PV 🡪 IL-6 | .035** | .013 | .008, .060 |
| IL-6 🡪 Internalising | .318*** | .091 | .143, .497 |
| Indirect effect | .011** | .005 | .003, .025 |
| Total effect | .569*** | .089 | .392, .738 |
| ***Adjusted model*** | | | |
| PV 🡪 Internalising | .479*** | .080 | .306, .649 |
| PV 🡪 IL-6 | .028* | .013 | .003, .053 |
| IL-6 🡪 Internalising | .147 | .092 | -.026, .320 |
| Indirect effect | .004 | .003 | .000, .014 |
| Total effect | .483*** | .087 | .309, .653 |

Table S9. Unadjusted and adjusted unstandardised regression coefficients (life events 🡪 IL-6 🡪 internalising)

| **Life Events (LE) 🡪 IL-6 🡪 Internalising** | | | |
| --- | --- | --- | --- |
|  | **b** | **SE** | **95% CI** |
| ***Unadjusted model*** | | | |
| LE 🡪 Internalising | .277*** | .035 | .195, .359 |
| LE 🡪 IL-6 | .017** | .006 | .006, .028 |
| IL-6 🡪 Internalising | .318*** | .091 | .143, .497 |
| Indirect effect | .005** | .002 | .002, .011 |
| Total effect | .283*** | .042 | .392, .738 |
| ***Adjusted model*** | | | |
| LE 🡪 Internalising | .201*** | .034 | .120, .282 |
| LE 🡪 IL-6 | .005 | .005 | -.005, .017 |
| IL-6 🡪 Internalising | .147 | .092 | -.026, .320 |
| Indirect effect | .001 | .001 | .000, .004 |
| Total effect | .202*** | .041 | .121, .292 |

Table S10. Unadjusted and adjusted unstandardised regression coefficients (peer victimisation 🡪 CRP 🡪 internalising)

| **Peer Victimisation (PV) 🡪 CRP 🡪 Internalising** | | | |
| --- | --- | --- | --- |
|  | **b** | **SE** | **95% CI** |
| ***Unadjusted model*** | | | |
| PV 🡪 Internalising | .564*** | .082 | .387, .734 |
| PV 🡪 CRP | .043* | .018 | .005, .081 |
| CRP 🡪 Internalising | .237*** | .067 | .103, .379 |
| Indirect effect | .010** | .006 | .002, .025 |
| Total effect | .575*** | .089 | .398, .744 |
| ***Adjusted model*** | | | |
| PV 🡪 Internalising | .482*** | .080 | .309, .651 |
| PV 🡪 CRP | .029 | .016 | -.004, .062 |
| CRP 🡪 Internalising | .099 | .074 | -.045, .250 |
| Indirect effect | .003 | .003 | -.001, .013 |
| Total effect | .485*** | .087 | .312, .656 |

Table S11. Unadjusted and adjusted unstandardised regression coefficients (life events 🡪 CRP 🡪 internalising)

| **Life Events (LE) 🡪 CRP 🡪 Internalising** | | | |
| --- | --- | --- | --- |
|  | **b** | **SE** | **95% CI** |
| ***Unadjusted model*** | | | |
| LE 🡪 Internalising | .282*** | .035 | .199, .364 |
| LE 🡪 CRP | .003 | .008 | -.014, .018 |
| CRP 🡪 Internalising | .237*** | .067 | .103, .379 |
| Indirect effect | .001 | .002 | -.003, .005 |
| Total effect | .283*** | .042 | .200, .365 |
| ***Adjusted model*** | | | |
| LE 🡪 Internalising | .206*** | .034 | .124, .286 |
| LE 🡪 CRP | -.017 | .007 | -.031, -.003 |
| CRP 🡪 Internalising | .099 | .074 | -.045, .250 |
| Indirect effect | -.002 | .002 | -.006, .000 |
| Total effect | .204*** | .041 | .122, .284 |

Table S12. Unadjusted and adjusted unstandardised regression coefficients (peer victimisation 🡪 IL-6 🡪 hyperactivity)

| **Peer Victimisation (PV) 🡪 IL-6 🡪 Hyperactivity** | | | |
| --- | --- | --- | --- |
|  | **b** | **SE** | **95% CI** |
| ***Unadjusted model*** | | | |
| PV 🡪 Hyperactivity | .519*** | .070 | .376, .667 |
| PV 🡪 IL-6 | .032* | .013 | .005, .057 |
| IL-6 🡪 Hyperactivity | -.136 | .078 | -.292, .31 |
| Indirect effect | -.004 | .003 | -.013, .000 |
| Total effect | .515*** | .074 | .371, .662 |
| ***Adjusted model*** | | | |
| PV 🡪 Hyperactivity | .461*** | .067 | .324, .602 |
| PV 🡪 IL-6 | .028* | .013 | .002, .052 |
| IL-6 🡪 Hyperactivity | -.087 | .077 | -.243, .076 |
| Indirect effect | -.002 | .003 | -.010, .001 |
| Total effect | .459*** | .072 | .321, .598 |

Table S13. Unadjusted and adjusted unstandardised regression coefficients (life events 🡪 IL-6 🡪 hyperactivity)

| **Life Events (LE) 🡪 IL-6 🡪 Hyperactivity** | | | |
| --- | --- | --- | --- |
|  | **b** | **SE** | **95% CI** |
| ***Unadjusted model*** | | | |
| LE 🡪 Hyperactivity | .125*** | .030 | .064, .191 |
| LE 🡪 IL-6 | .009 | .006 | -.002, .021 |
| IL-6 🡪 Hyperactivity | -.136 | .078 | -.292, .31 |
| Indirect effect | -.001 | .001 | -.005, .000 |
| Total effect | .124*** | .032 | .063, .190 |
| ***Adjusted model*** | | | |
| LE 🡪 Hyperactivity | .122*** | .028 | .064, .184 |
| LE 🡪 IL-6 | .006 | .005 | -.005, .017 |
| IL-6 🡪 Hyperactivity | -.087 | .077 | -.243, .76 |
| Indirect effect | -.001 | .001 | -.003, .000 |
| Total effect | .121*** | .031 | .064, .184 |

Table S14. Unadjusted and adjusted unstandardised regression coefficients (peer victimisation 🡪 CRP 🡪 hyperactivity)

| **Peer Victimisation (PV) 🡪 CRP 🡪 Hyperactivity** | | | |
| --- | --- | --- | --- |
|  | **b** | **SE** | **95% CI** |
| ***Unadjusted model*** | | | |
| PV 🡪 Hyperactivity | .561*** | .070 | .415, .710 |
| PV 🡪 CRP | .044* | .018 | .006, .081 |
| CRP 🡪 Hyperactivity | -.190*** | .057 | -.302, -.075 |
| Indirect effect | -.008* | .005 | -.020, -.002 |
| Total effect | .553*** | .075 | .408, .700 |
| ***Adjusted model*** | | | |
| PV 🡪 Hyperactivity | .461*** | .067 | .322, .600 |
| PV 🡪 CRP | .030 | .016 | -.003, .063 |
| CRP 🡪 Hyperactivity | -.029 | .062 | -.157, .093 |
| Indirect effect | -.001 | .002 | -.008, .002 |
| Total effect | .460*** | .072 | .322, .599 |

Table S15. Unadjusted and adjusted unstandardised regression coefficients (life events 🡪 CRP 🡪 hyperactivity)

| **Life Events (LE) 🡪 CRP 🡪 Hyperactivity** | | | |
| --- | --- | --- | --- |
|  | **b** | **SE** | **95% CI** |
| ***Unadjusted model*** | | | |
| LE 🡪 Hyperactivity | .147*** | .030 | .085, .212 |
| LE 🡪 CRP | .004 | .008 | -.012, .019 |
| CRP 🡪 Hyperactivity | -.190*** | .057 | -.302, -.075 |
| Indirect effect | -.001 | .002 | -.004, .002 |
| Total effect | .146*** | .032 | .085, .211 |
| ***Adjusted model*** | | | |
| LE 🡪 Hyperactivity | .122*** | .028 | .064, .185 |
| LE 🡪 CRP | -.016* | .007 | -.030, -.003 |
| CRP 🡪 Hyperactivity | -.029 | .062 | -.157, .093 |
| Indirect effect | .000 | .001 | -.001, .003 |
| Total effect | .122*** | .031 | .064, .185 |

Table S16. Unadjusted and adjusted unstandardised regression coefficients (peer victimisation 🡪 IL-6 🡪 conduct problems)

| **Peer Victimisation (PV) 🡪 IL-6 🡪 Conduct Problems (CP)** | | | |
| --- | --- | --- | --- |
|  | **b** | **SE** | **95% CI** |
| ***Unadjusted model*** | | | |
| PV 🡪 CP | .208*** | .046 | .112, .305 |
| PV 🡪 IL-6 | .035** | .013 | .008, .060 |
| IL-6 🡪 CP | .097 | .051 | .006, .188 |
| Indirect effect | .003* | .002 | .000, .010 |
| Total effect | .212*** | .049 | .115, .308 |
| ***Adjusted model*** | | | |
| PV 🡪 CP | .161*** | .044 | .066, .253 |
| PV 🡪 IL-6 | .028* | .013 | .003, .053 |
| IL-6 🡪 CP | .025 | .051 | -.069, .117 |
| Indirect effect | .001 | .002 | -.002, .005 |
| Total effect | .162*** | .048 | .068, .255 |

Table S17. Unadjusted and adjusted unstandardised regression coefficients (life events 🡪 IL-6 🡪 conduct problems)

| **Life Events (LE) 🡪 IL-6 🡪 Conduct Problems (CP)** | | | |
| --- | --- | --- | --- |
|  | **b** | **SE** | **95% CI** |
| ***Unadjusted model*** | | | |
| LE 🡪 CP | .082*** | .019 | .040, .124 |
| LE 🡪 IL-6 | .017** | .006 | .007, .029 |
| IL-6 🡪 CP | .097 | .051 | .006, .188 |
| Indirect effect | .002* | .001 | .000, .004 |
| Total effect | .083*** | .021 | .042, .125 |
| ***Adjusted model*** | | | |
| LE 🡪 CP | .037 | .019 | -.004, .077 |
| LE 🡪 IL-6 | .006 | .005 | -.005, .017 |
| IL-6 🡪 CP | .025 | .051 | -.069, .117 |
| Indirect effect | .000 | .000 | .000, .002 |
| Total effect | .037 | .021 | -.004, .077 |

Table S18. Unadjusted and adjusted unstandardised regression coefficients (peer victimisation 🡪 CRP 🡪 conduct problems)

| **Peer Victimisation (PV) 🡪 CRP 🡪 Conduct Problems (CP)** | | | |
| --- | --- | --- | --- |
|  | **b** | **SE** | **95% CI** |
| ***Unadjusted model*** | | | |
| PV 🡪 CP | .213*** | .046 | .116, .310 |
| PV 🡪 CRP | .045* | .018 | .007, .082 |
| CRP 🡪 CP | .018 | .038 | -.056, .095 |
| Indirect effect | .001 | .002 | -.002, .006 |
| Total effect | .214*** | .049 | .117, .311 |
| ***Adjusted model*** | | | |
| PV 🡪 CP | .164*** | .044 | .069, .256 |
| PV 🡪 CRP | .030 | .016 | -.003, .063 |
| CRP 🡪 CP | -.024 | .041 | -.099, .059 |
| Indirect effect | -.001 | .001 | -.005, .001 |
| Total effect | .163*** | .048 | .069, .256 |

Table S19. Unadjusted and adjusted unstandardised regression coefficients (life events 🡪 CRP 🡪 conduct problems)

| **Life Events (LE) 🡪 CRP 🡪 Conduct Problems (CP)** | | | |
| --- | --- | --- | --- |
|  | **b** | **SE** | **95% CI** |
| ***Unadjusted model*** | | | |
| LE 🡪 CP | .083*** | .019 | .042, .125 |
| LE 🡪 CRP | .003 | .008 | -.013, .019 |
| CRP 🡪 CP | .018 | .038 | -.056, .095 |
| Indirect effect | .000 | .000 | .000, .001 |
| Total effect | .083*** | .021 | .042, .126 |
| ***Adjusted model*** | | | |
| LE 🡪 CP | .037 | .019 | -.004, .078 |
| LE 🡪 CRP | -.017* | .007 | -.030, -.003 |
| CRP 🡪 CP | -.024 | .041 | -.099, .059 |
| Indirect effect | .000 | .001 | -.001, .002 |
| Total effect | .037 | .021 | -.003, .078 |

Table S20. Unadjusted and adjusted unstandardised regression coefficients (peer victimisation 🡪 IL-6 🡪 externalising)

| **Peer Victimisation (PV) 🡪 IL-6 🡪 Externalising** | | | |
| --- | --- | --- | --- |
|  | **b** | **SE** | **95% CI** |
| ***Unadjusted model*** | | | |
| PV 🡪 Externalising | .766*** | .101 | .558, .979 |
| PV 🡪 IL-6 | .034* | .013 | .007, .060 |
| IL-6 🡪 Externalising | -.072 | .111 | -.287, .144 |
| Indirect effect | -.002 | .004 | -.013, .004 |
| Total effect | .764*** | .107 | .555, .974 |
| ***Adjusted model*** | | | |
| PV 🡪 Externalising | .625*** | .096 | .427, .827 |
| PV 🡪 IL-6 | .028* | .013 | .002, .052 |
| IL-6 🡪 Externalising | -.080 | .110 | -.298, .135 |
| Indirect effect | -.002 | .003 | -.012, .003 |
| Total effect | .622*** | .102 | .425, .823 |

Table S21. Unadjusted and adjusted unstandardised regression coefficients (life events 🡪 IL-6 🡪 externalising)

| **Life Events (LE) 🡪 IL-6 🡪 Externalising** | | | |
| --- | --- | --- | --- |
|  | **b** | **SE** | **95% CI** |
| ***Unadjusted model*** | | | |
| LE 🡪 Externalising | .229*** | .043 | .144, .319 |
| LE 🡪 IL-6 | .017** | .006 | .007, .029 |
| IL-6 🡪 Externalising | -.072 | .111 | -.287, .144 |
| Indirect effect | -.001 | .002 | -.006, .002 |
| Total effect | .228*** | .045 | .142, .318 |
| ***Adjusted model*** | | | |
| LE 🡪 Externalising | .157*** | .040 | .077, .245 |
| LE 🡪 IL-6 | .006 | .005 | -.005, .017 |
| IL-6 🡪 Externalising | -.080 | .110 | -.298, .135 |
| Indirect effect | .000 | .001 | -.004, .001 |
| Total effect | .157*** | .043 | .077, .244 |

Table S22. Unadjusted and adjusted unstandardised regression coefficients (peer victimisation 🡪 CRP 🡪 externalising)

| **Peer Victimisation (PV) 🡪 CRP🡪 Externalising** | | | |
| --- | --- | --- | --- |
|  | **b** | **SE** | **95% CI** |
| ***Unadjusted model*** | | | |
| PV 🡪 Externalising | .773*** | .101 | .563, .985 |
| PV 🡪 CRP | .044* | .018 | .006, .082 |
| CRP 🡪 Externalising | -.180* | .082 | -.342, -.018 |
| Indirect effect | -.008* | .005 | -.023, -.001 |
| Total effect | .765*** | .107 | .556, .975 |
| ***Adjusted model*** | | | |
| PV 🡪 Externalising | .627*** | .096 | .429, .828 |
| PV 🡪 CRP | .030 | .016 | -.003, .063 |
| CRP 🡪 Externalising | -.065 | .089 | -.237, .107 |
| Indirect effect | -.002 | .003 | -.011, .002 |
| Total effect | .625*** | .102 | .427, .826 |

Table S23. Unadjusted and adjusted unstandardised regression coefficients (life events 🡪 CRP 🡪 externalising)

| **Life Events (LE) 🡪 CRP🡪 Externalising** | | | |
| --- | --- | --- | --- |
|  | **b** | **SE** | **95% CI** |
| ***Unadjusted model*** | | | |
| LE 🡪 Externalising | .229*** | .042 | .144, .319 |
| LE 🡪 CRP | .004 | .008 | -.013, .019 |
| CRP 🡪 Externalising | -.180* | .082 | -.342, -.018 |
| Indirect effect | -.001 | .002 | -.005, .002 |
| Total effect | .229*** | .045 | .143, .319 |
| ***Adjusted model*** | | | |
| LE 🡪 Externalising | .157*** | .040 | .077, .245 |
| LE 🡪 CRP | -.017* | .007 | -.030, -.003 |
| CRP 🡪 Externalising | -.065 | .089 | -.237, .107 |
| Indirect effect | .001 | .002 | -.001, .006 |
| Total effect | .158*** | .043 | .079, .246 |
